# Alpha-defensin 5 differentially modulates adenovirus vaccine vectors from different serotypes *in vivo*

**DOI:** 10.1371/journal.ppat.1008180

**Published:** 2019-12-16

**Authors:** Lawrence J. Tartaglia, Alexander Badamchi-Zadeh, Peter Abbink, Eryn Blass, Malika Aid, Makda S. Gebre, Zhenfeng Li, Kevin Clyde Pastores, Sebastien Trott, Siddhant Gupte, Rafael A. Larocca, Dan H. Barouch

**Affiliations:** 1 Center for Virology and Vaccine Research, Beth Israel Deaconess Medical Center, Harvard Medical School, Boston Massachusetts, United States of America; 2 Ragon Institute of MGH, MIT, and Harvard, Cambridge, Massachusetts, United States of America; The Children's Hospital of Philadelphia, UNITED STATES

## Abstract

Adenoviral vectors have shown significant promise as vaccine delivery vectors due to their ability to elicit both innate and adaptive immune responses. α-defensins are effector molecules of the innate immune response and have been shown to modulate natural infection with adenoviruses, but the majority of α-defensin-adenovirus interactions studied to date have only been analyzed *in vitro*. In this study, we evaluated the role of α-defensin 5 (HD5) in modulating adenovirus vaccine immunogenicity using various serotype adenovirus vectors in mice. We screened a panel of human adenoviruses including Ad5 (species C), Ad26 (species D), Ad35 (species B), Ad48 (species D) and a chimeric Ad5HVR48 for HD5 sensitivity. HD5 inhibited transgene expression from Ad5 and Ad35 but augmented transgene expression from Ad26, Ad48, and Ad5HVR48. HD5 similarly suppressed antigen-specific IgG and CD8^+^ T cell responses elicited by Ad5 vectors in mice, but augmented IgG and CD8^+^ T cell responses and innate cytokine responses elicited by Ad26 vectors in mice. Moreover, HD5 suppressed the protective efficacy of Ad5 vectors but enhanced the protective efficacy of Ad26 vectors expressing SIINFEKL against a surrogate Listeria-OVA challenge in mice. These data demonstrate that HD5 differentially modulates adenovirus vaccine delivery vectors in a species-specific manner *in vivo*.

## Author summary

Previous studies have shown that human α-defensin 5 (HD5) modulates adenovirus gene expression *in vitro*. Species D and F adenoviruses are generally augmented in the presence of HD5, while most other adenoviruses are inhibited. However, data regarding Ad-HD5 interactions *in vivo* are limited. We therefore sought to elucidate the capacity of HD5 to modulate Ad vectors from various serotypes *in vivo*. We immunized mice with Ad5 or Ad26 vectors together with HD5. We observed that HD5 augmented Ad26-elicited innate, cellular, and humoral immune responses but suppressed Ad5 immunogenicity. This study demonstrates that HD5 regulates Ad vector immunogenicity *in vivo* and suggests potential therapeutic applications of HD5.

## Introduction

Human α-defensins are small, cationic peptides consisting of 18–45 amino acids folded into a β-sheet structure that is stabilized by three disulfide bonds. To date, six α-defensins have been identified in humans. Human neutrophil peptides (HNP) 1, 2, 3 and 4 are primarily secreted from neutrophils and participate in systemic innate immunity [1], whereas human defensins (HD) 5 and 6 are typically secreted from intestinal Paneth cells and contribute to gastrointestinal tract innate immunity [2]. α-defensins have also shown the ability to neutralize bacteria, fungi, and viral targets [3,4].

α-defensins exhibit potent antiviral activity against adenoviruses [5]. *In vitro* experiments have shown that α-defensins inhibit expression from adenoviruses from species A, B1, B2, C, and E, whereas α-defensins may augment expression from adenoviruses from species D and F [5,6]. However, it has remained unclear how α-defensins modulate adenoviruses *in vivo*. Mechanistically, α-defensins are believed to inhibit adenoviruses by preventing uncoating, thereby restricting release of the endosomolytic protein (protein VI) during endosomal escape [6]. Consequently, adenoviral virions accumulate in endosomal compartments and fail to reach the nucleus. Although the precise mechanism of how α-defensins augment gene expression by species D and F adenoviruses is not fully understood, it has been proposed that increased numbers of Ad-defensin complexes may accumulate at cell surfaces [7] and may be internalized through defensin-specific receptors [8].

Several studies have suggested that enteric α-defensins enhance infectivity of mouse adenovirus types 1 and 2 in mice [8,9]. Furthermore, α-defensins have been shown to exert adjuvant-like properties when co-administered with antigens [10–15]. However, the potential impact of human α-defensins on human adenovirus vaccine vectors has not previously been studied *in vivo* and is important for defining the potential therapeutic use of α-defensins. We hypothesized that α-defensins, and in particular α-defensin 5 (HD5), may differentially modulate the immunogenicity of adenovirus vaccine vectors from different serotypes.

In this study, we assessed whether HD5 would modulate transgene expression from adenovirus serotypes 5, 26, 35, 48 (Ad5, Ad26, Ad35, Ad48) and a chimeric Ad5 vector containing the surface hexon hypervariable regions of Ad48 (Ad5HVR48), and whether HD5 would modulate immunogenicity by Ad5 and Ad26 vaccine vectors in mice. Co-administration of HD5 with Ad5 suppressed transgene expression, immunogenicity, and protective efficacy. In contrast, co-administration of HD5 with Ad26 enhanced transgene expression, increased Ad26-elicited innate cytokines, antibody, and CD8^+^ T cells responses, and improved protective efficacy against a recombinant Listeria challenge.

## Results

### HD5 alters adenovirus transduction sensitivity in A549 cells

**Fig 1:**
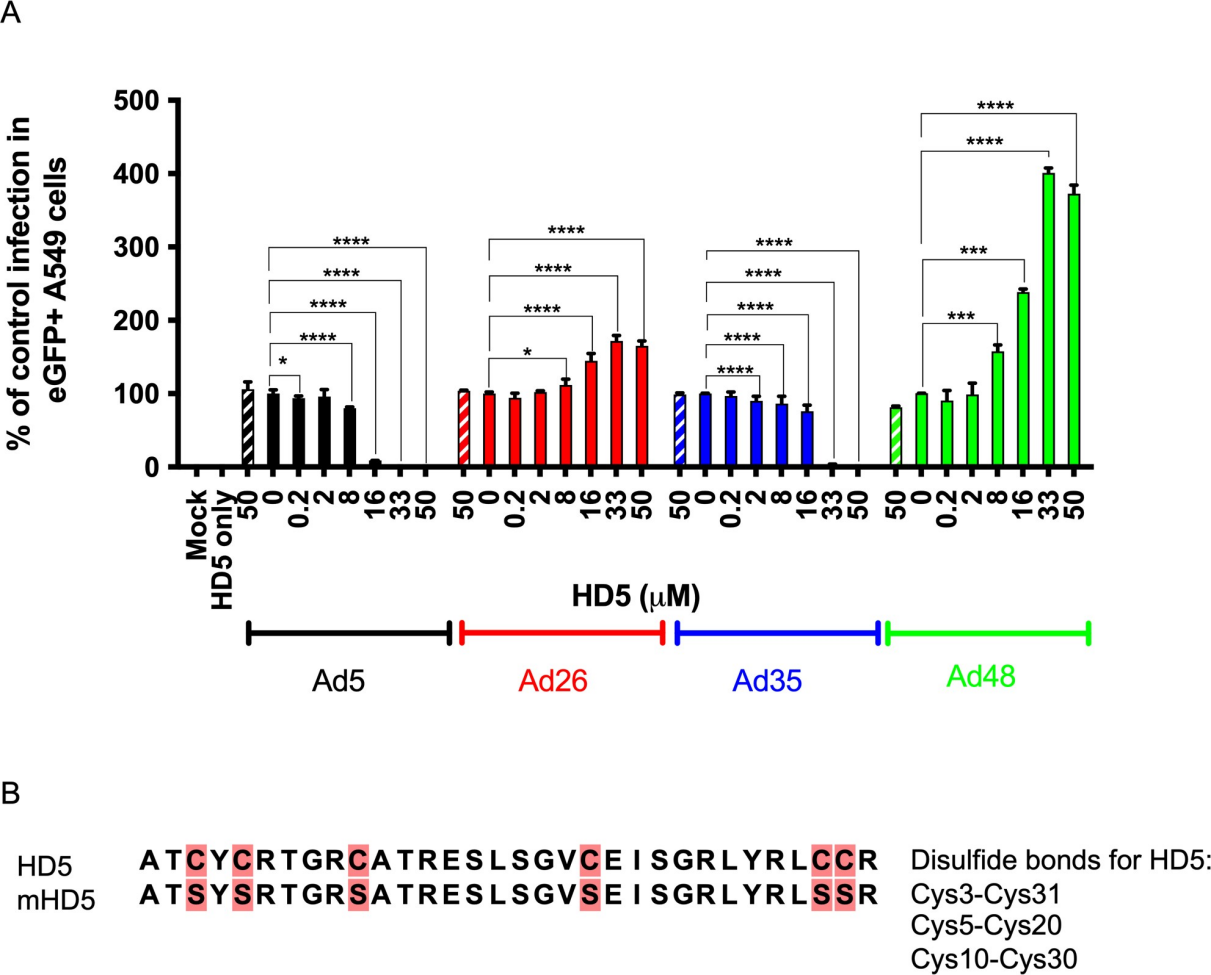
Analysis of adenovirus sensitivity to HD5 in A549 cells. Ad5, Ad26, Ad35, and Ad48 were incubated with 0.2 μM– 50 μM HD5 or 50 μM mutant HD5 (diagonal box) and assessed for % of cells expressing eGFP 24 h post infection. Experimental results are normalized to control infected with virus (100%) in the absence of peptide. (B) HD5 and mHD5 amino acid sequence alignment. Cysteine → Serine mutations are highlighted in pink. Data is expressed as the mean (±SD) of three independent experiments. **** *p* < 0.0001, *** *p* < 0.001, ** *p* < 0.01, * *p* < 0.05, one-way ANOVA test (compared to virus only control).

Previous *in vitro* studies have shown that HD5 inhibits most adenovirus types, except adenoviruses from species D and F [5]. Based on these data, we postulated that HD5 might exhibit different effects with different adenovirus vectors *in vivo*. We studied two species D adenoviruses, Ad26 and Ad48 [16], expressing enhanced Green Fluorescent Protein (eGFP) for an initial *in vitro* study. For comparison, Ad5.eGFP (species C) and Ad35.eGFP (species B) were also included. We selected A549 cells for initial tests with HD5 as A549 cells are often used to study Ad infectivity [3,5,6,17]. Control infection was normalized to 100% of eGFP-positive cells 24 h post-infection in the absence of peptide. Ad5.eGFP was ≥99% inhibited at a concentration of ≥16 μM HD5 (*p* < 0.0001), and Ad35.eGFP showed 100% inhibition at a HD5 concentration of ≥33 μM (*p* < 0.0001) compared to control infection (Fig 1A). In contrast, transduction of Ad26.eGFP and Ad48.eGFP was enhanced at concentrations of ≥8 μM HD5 (Ad26, *p* < 0.05; Ad48, *p* < 0.0001) (Fig 1A). Peak expression was at 72% (*p* < 0.0001) above control infection for Ad26.eGFP and 400% (*p* < 0.0001) above control infection for Ad48.eGFP with 33 μM HD5. In contrast, a negative control mutant HD5 peptide (Fig 1B) (mHD5; 50 μM) did not modulate adenovirus infection compared to wild type (WT) infection (no HD5) for all viruses as expected (Fig 1A). Taken together, these data show that transgene expression from Ad26 and Ad48 vectors was enhanced by HD5 pre-treatment [6].

**Fig 2:**
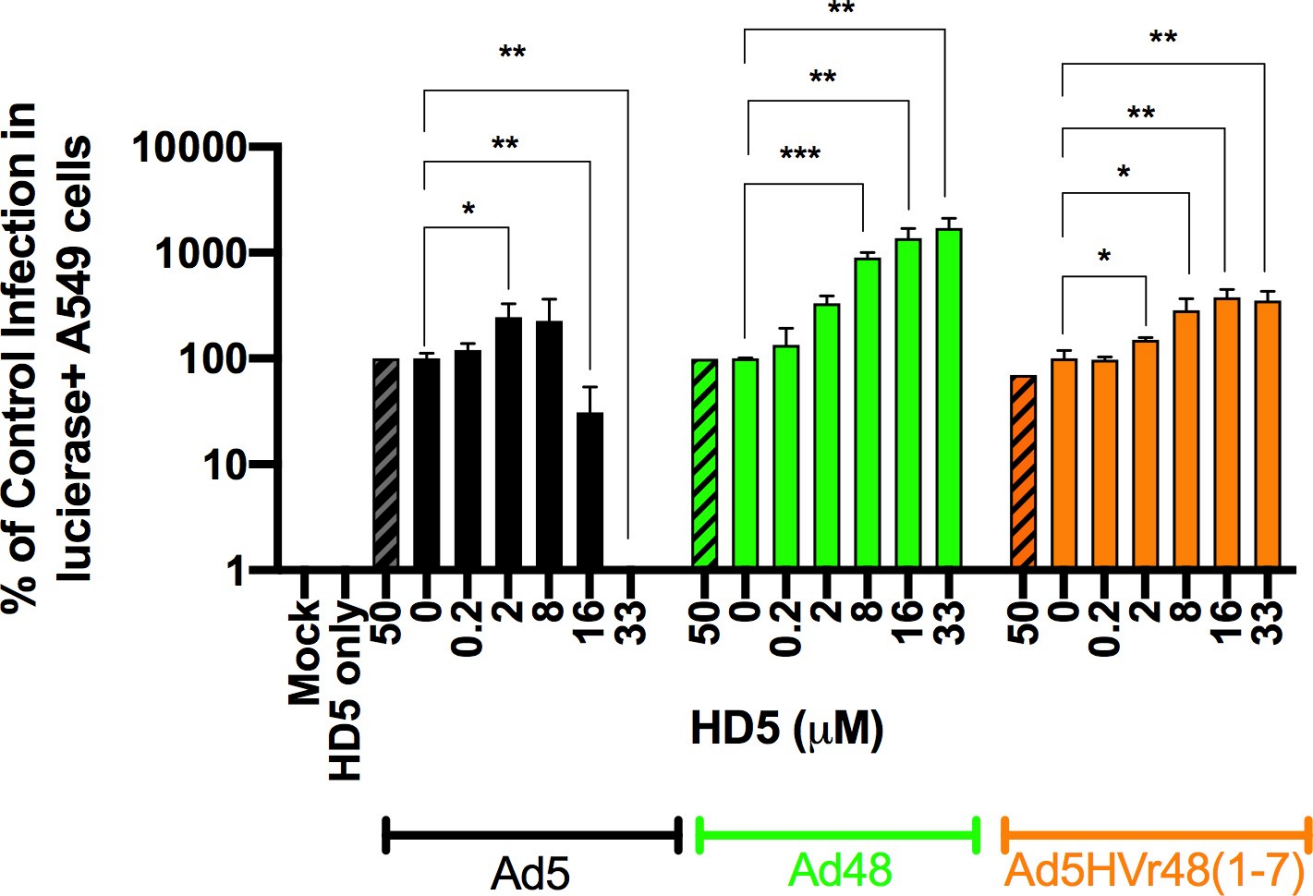
Analysis of Ad5HVR48 sensitivity to HD5 in A549 cells. Ad5, Ad48, and Ad5HVR48 were incubated with 0.2 μM– 33 μM HD5 or 50 μM mutant HD5 (diagonal box) and assessed for % of cells expressing luciferase 24 h post infection. Experimental results are normalized to control infected with virus (100%) in the absence of peptide. Data is expressed as the mean (±SD) of three independent experiments. *** *p* < 0.001, ** *p* < 0.01, * *p* < 0.05, one-way ANOVA test (compared to virus only control).

Prior studies have suggested that HD5 can bind to both Ad fiber and hexon proteins [5,18]. To evaluate potential interactions between HD5 and species D adenoviruses, we assessed the ability of HD5 to modulate the infectivity of a chimeric Ad5HVR48 virus, which is 99% Ad5 and contains the Ad5 fiber but has the surface hexon hypervariable regions (HVR) of Ad48 [19–21]. Chimeric Ad5/Ad26 vectors proved unstable and could not be constructed. Control infection in A549 cells was normalized to 100% of luciferase-positive cells 24 h post-infection in the absence of peptide. As shown in the previous experiment, expression from Ad5.luc was abrogated with 33 μM HD5 (*p* < 0.05), but expression from both Ad48.luc and Ad5HVR48.luc were markedly enhanced in the presence of HD5 (*p* < 0.01) (Fig 2). Since Ad5HVR48 shares the phenotype of Ad48, these data suggest that HD5 can interact with the surface hexon HVRs of Ad48, although we cannot exclude the possibility that HD5 may also interact with other Ad capsid proteins.

### HD5 impacts adenovirus expression *in vivo*

**Fig 3:**
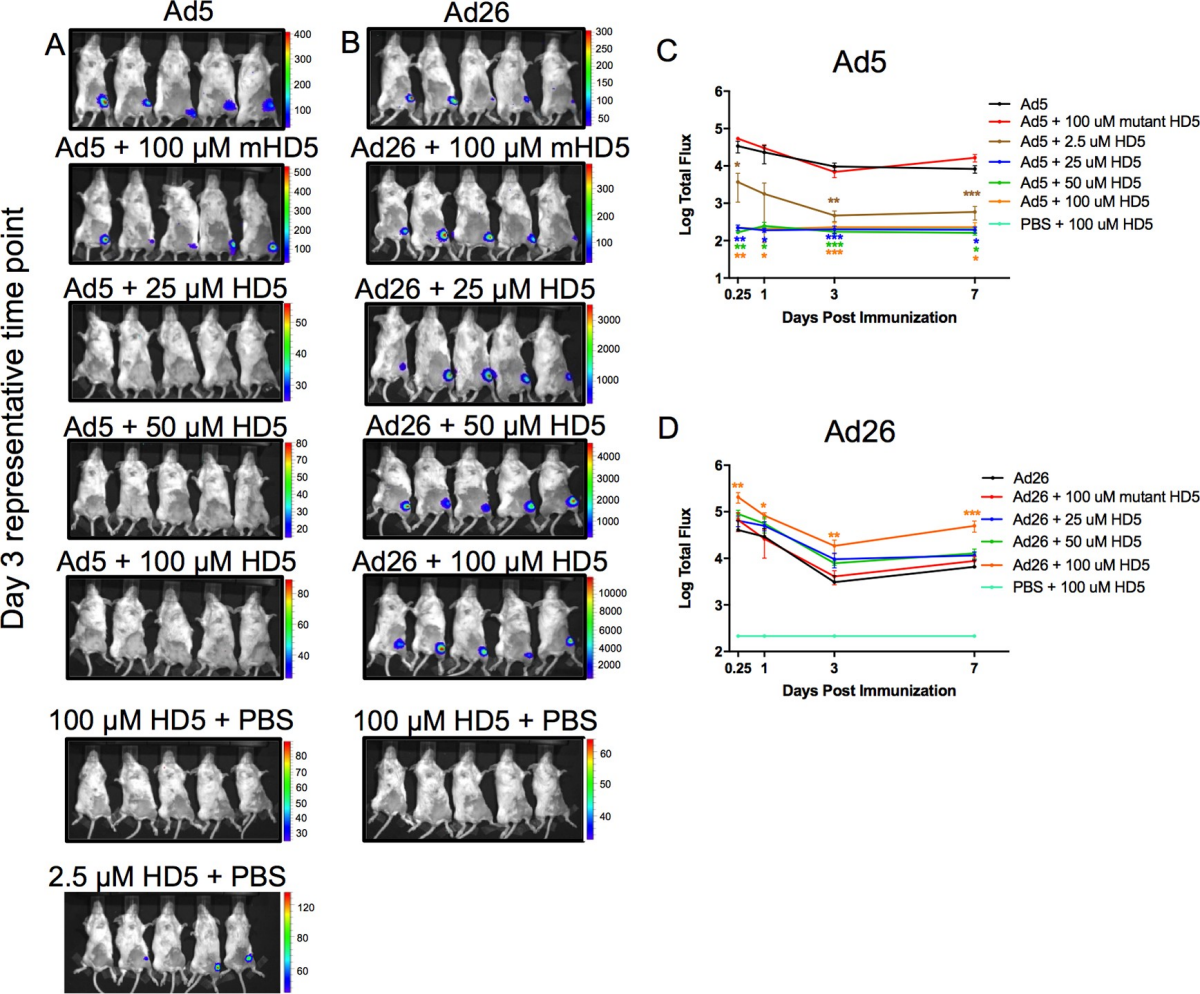
In vivo luciferase transgene expression by Ad5 and Ad26 ± HD5 pretreatment. Animals were pretreated with a dose titration of 2.5 μM– 100 μM HD5, 100 μM mutant HD5 (linear peptide), or PBS ± 100 μM HD5 and immunized i.m. with (A) Ad5 (10^9^ vp) or (B) 25 μM– 100 μM HD5 with Ad26 (10^9^ vp) vectors encoding luciferase transgene cassettes. Representative day 3 IVIS images are shown for each experimental condition assayed. The Total Flux (photons/sec/cm^2^/radian) released by luciferase activity was averaged for (C) Ad5 and (D) Ad26 at each time-point. n = 5 animals per group. The data are representative of experiments performed two times. Mean ± SEM are shown. *** *p* < 0.001, ** *p* < 0.01, * *p* < 0.05, one-way ANOVA test (compared to virus only control).

We next immunized BALB/C mice with adenovirus vectors from different serotypes expressing luciferase to evaluate transgene expression *in vivo*. Ad5.luc (10^9^ vp) and Ad26.luc (10^9^ vp) were administered by the intramuscular (i.m.) route, and luciferase expression was monitored following intraperitoneal (i.p.) injection of luciferin substrate. Expression was assessed at 6 h, 1, 3, and 7 days post immunization. Animals that received Ad5.luc and 2.5 μM HD5 showed a 21-fold reduction in expression on day 3 (*p* < 0.001), whereas concentrations of ≥25 μM HD5 showed no detectable expression (Fig 3A and 3C). In contrast, a 3-fold to 8-fold enhanced luciferase expression was observed in animals receiving Ad26.luc and ≥25 μM HD5 (100 μM, *p* < 0.001 at day 7) (Fig 3B and 3D). As expected, the negative control mutant mHD5 showed no significant impact on any of the experimental groups assayed (Fig 3). These *in vivo* observations mirror the *in vitro* data (Fig 1A), which showed HD5 pre-treatment inhibits Ad5 and augments Ad26 transgene expression.

### HD5 differentially drives Ad-elicited innate immune responses *in vivo*

**Fig 4:**
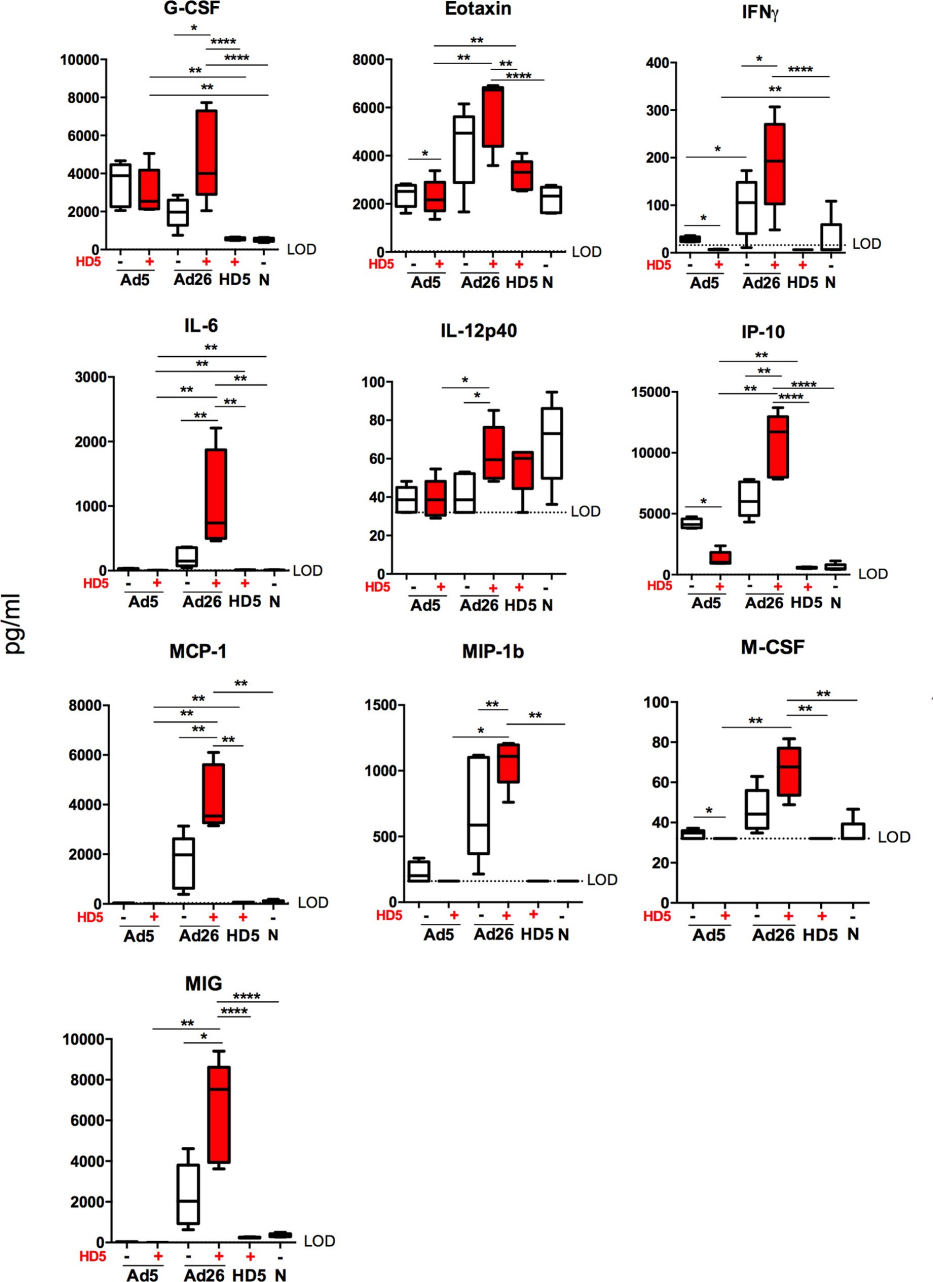
Ad5- and Ad26-elicited cytokine and chemokine responses in mice. C57BL/6 mice (n = 5/group) were administered i.m. with either PBS or 100 μM HD5 and Ad5 (10^10^ vp) or Ad26 (10^10^ vp) empty vectors. Box plots for selected cytokines that show statistical significance among groups. The data are representative of experiments performed two times. Mean ± SEM and limit of detection (LOD) are shown. ****p < 0.0001, ***p < 0.001, ** *p* < 0.01, * *p* < 0.05, Wilcox statistical test.

To characterize the early innate immune cytokine profiles elicited by Ad5 and Ad26 vectors, we immunized C57BL/6 mice i.m. with Ad5 (10^10^ vp) and Ad26 (10^10^ vp) empty vectors with or without 100 μM HD5 pretreatment. Sera was isolated from animals at 7 h post immunization [22] and analyzed by Luminex assays as previously described [23]. HD5 induced elevated innate cytokine and chemokine responses in animals treated with Ad26 and decreased innate cytokine and chemokine responses in animals treated with Ad5 (Fig 4). Ad26 pretreated with HD5 induced significantly higher levels than Ad26 alone of granulocyte colony stimulating factor (G-CSF, p < 0.05), interferon gamma (IFN-γ, p < 0.05), interleukin-6 (IL-6, p < 0.01), interleukin-12 p40 (IL-12 p40, p < 0.05), interferon gamma-induced protein (IP-10, p < 0.01), monocyte chemotactic protein 1 (MCP-1, p < 0.01), macrophage inflammatory protein 1b (MIP-1b, p < 0.01), and monokine induced by gamma interferon (MIG, p < 0.05) (Fig 4). In contrast, HD5 pretreated Ad5 vectors induced significantly lower levels of Eotaxin (p < 0.05), IFN-γ (p < 0.01), IP-10 (p < 0.01), and M-CSF (p < 0.05) (Fig 4). These data suggest that HD5 differentially impacted Ad5- and Ad26-elicted innate cytokine profiles in mice.

### HD5 differentially impacts Ad-elicited antibody responses *in vivo*

**Fig 5:**
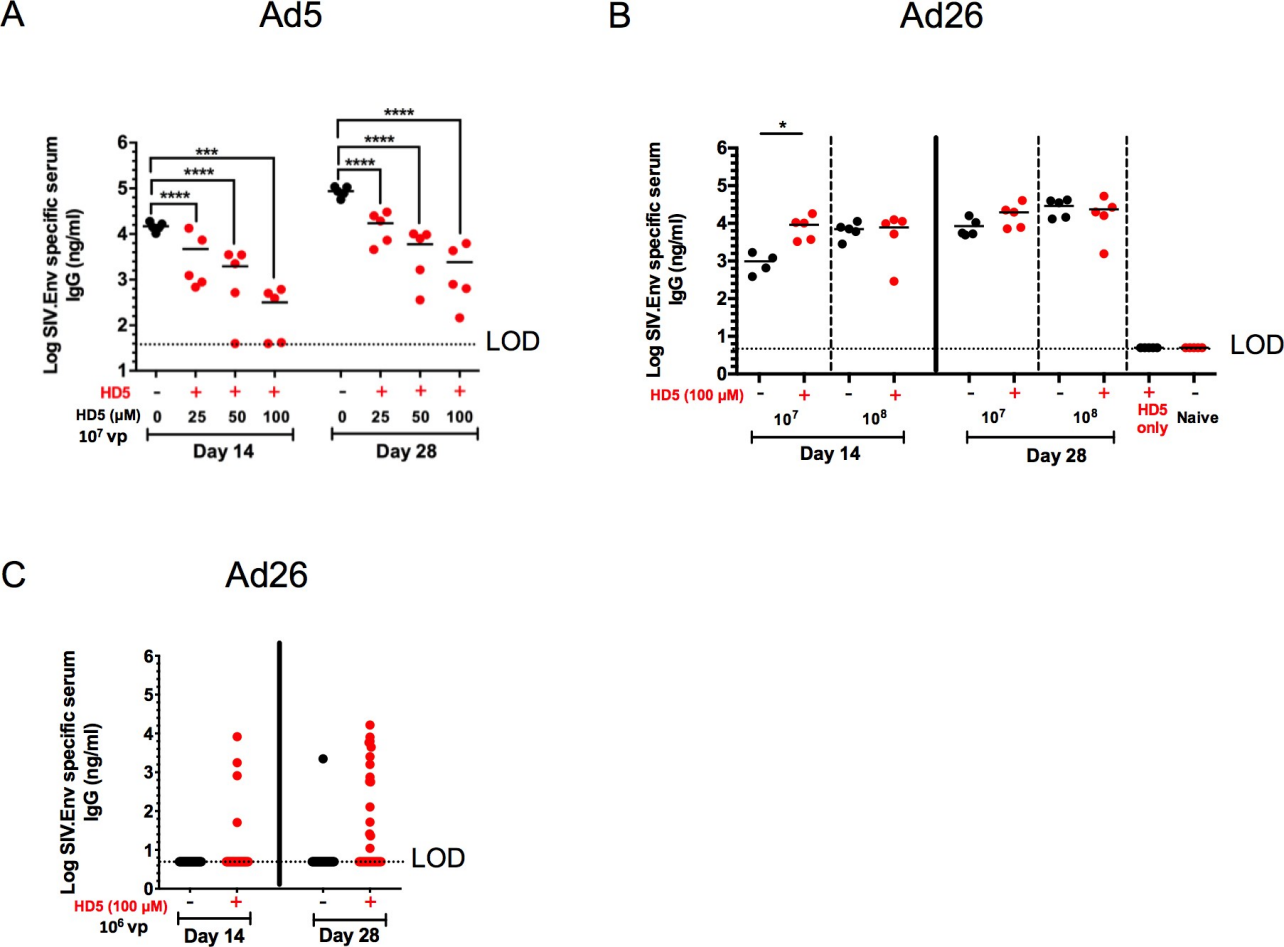
Vaccine-elicited antibody responses to Ad5 and Ad26 ± HD5 pretreatment. C57BL/6 mice (n = 5/group) were pretreated with either PBS or a dose titration of 25 μM– 100 μM HD5 with (A) Ad5.SIVEnv (10^9^ vp) or 100 μM HD5 with (B) Ad26.SIVEnv (10^8^–10^7^ vp) encoding SIV ENV. Env-specific IgG responses were determined in serum by ELISA at days 14 and 28 post immunization. Means and standard deviations of endpoint ELISA titers are shown. *** *p* < 0.001, ** *p* < 0.01, * *p* < 0.05, one-way ANOVA test (compared to virus only control). (C) 35 animals/group immunized with Ad26.SIVEnv (10^6^ vp) with and without 100 μM HD5 on Day 14 and 28 post immunization. Groups (Ad26-HD5 compared to Ad26) were assessed for significance on day 14 or day 28 using a Fisher Exact Test to identify nonrandom associations between groups. The day 28 analysis resulted in a two-tailed Fisher Exact Test p value = 0.0017. The data are representative of experiments performed three times.

We next evaluated whether HD5 administration would modulate the immunogenicity of Ad5 and Ad26 vaccine vectors in mice. Previous research has shown that defensins are capable of directly binding microbial antigens and forming complexes that may be more readily taken up by antigen-presenting cells, which effectively enhances the production of antigen-specific antibodies [24]. We therefore investigated whether HD5 impacted Env-specific IgG titers elicited by Ad5 and Ad26 vectors expressing SIV envelope protein (Env). Mice immunized with Ad5.SIVEnv (10^9^ vp) showed a 1.7 log reduction in Env-specific IgG titers by day 14 (100 μM HD5; p < 0.0001) and a 1.6 log reduction by day 28 (100 μM HD5; p < 0.0001) with 100 μM HD5 compared with controls (Fig 5A). In contrast, for Ad26.SIVEnv we observed a 9.3-fold enhancement of Env-specific IgG levels at day 14 (10^7^ vp, p < 0.05; Fig 5B). These results indicate that HD5 suppressed humoral immune responses induced by Ad5.SIVEnv but augmented humoral immune responses induced by Ad26.SIVEnv.

We next immunized large groups of animals (n = 35/group) with a low dose (10^6^) of Ad26.SIVEnv to evaluate if HD5 can boost humoral immune responses to a low vaccine dose. Fig 5C shows that 4 of 35 animals immunized with Ad26.SIVEnv and 100 μM HD5 had detectable Env-specific IgG titers on day 14, while no responses were detected in animals immunized with Ad26.SIVEnv alone. By day 28, 12 of 35 animals immunized with Ad26 and 100 μM HD5 had detectable Env-specific IgG titers, while only 1 of 34 animals immunized with Ad26.SIVEnv alone had detectable Env-specific IgG titers (Fig 5C; p = 0.0017, two-tailed Fisher’s exact test). These data suggest that HD5 can boost humoral immune responses at low Ad26 vector doses.

### HD5 differentially impacts Ad-elicited CD8^+^ T cell responses *in vivo*

**Fig 6:**
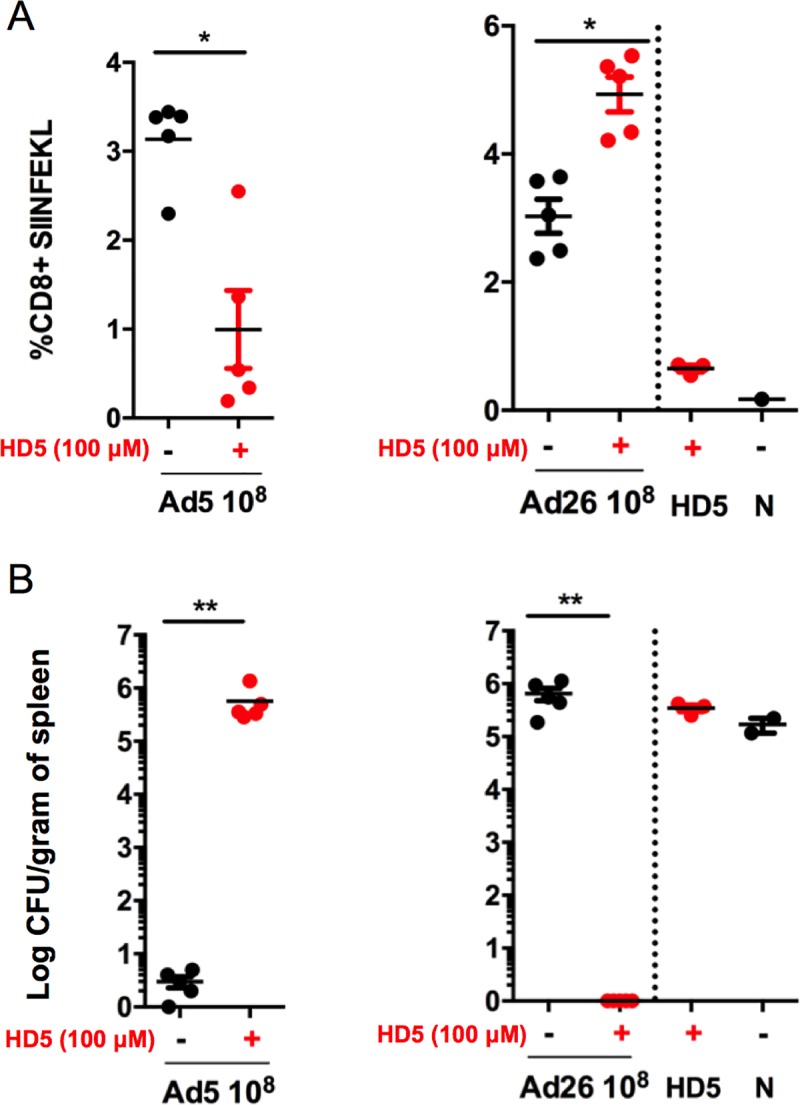
HD5 alters antigen-specific CD8^+^ T cell responses primed by Ad5 and Ad26 vectors in a recombinant *Listeria monocytogenes* challenge model in mice. C57BL/6 mice (n = 5/group) were administered i.m. Ad5.SIINFEKL (10^8^ vp) or Ad26.SIINFEKL (10^8^ vp) pretreated with either PBS or 100 μM HD5. (A) On day 11 after immunization, mice were bled and SIINFEKL-specific CD8+ T cell responses were measured by H-2K^b^ tetramer staining. Following bleeding, animals were challenged with 1.0 X 10^5^ CFU *Lm*-OVA. (B) The mean number of bacteria per spleen was determined 48 h after *Listeria monocytogenes* infection. The data are representative of experiments performed two times. Mean ± SEM are shown. ** *p* < 0.01, * *p* < 0.05, Mann-Whitney U Test (compared to virus only control).

We next assessed the effect of HD5 pretreatment on vaccine-elicited CD8^+^ T cell responses primed by Ad5 and Ad26 vectors in the context of a recombinant *Listeria monocytogenes*-ovalbumin expressed (*Lm-*OVA) challenge model. We immunized C57BL/6 mice i.m. with 10^8^ vp Ad5.SIINFEKL or Ad26.SIINFEKL vectors expressing the immunodominant ovalbumin epitope (SIINFEKL) and 100 μM HD5. Animals were bled 11 days post-immunization, and CD8^+^ T cell responses specific for SIINFEKL were evaluated by tetramer binding assays. Mice immunized with 100 μM HD5 and Ad5.SIINFEKL showed a 3.1-fold reduction in the mean percentage of SIINFEKL^+^ CD8^+^ T cells compared to the controls (p < 0.05; Fig 6A), whereas 100 μM HD5 led to a 1.6-fold enhancement of SIINFEKL^+^ CD8^+^ T cells elicited by Ad26.SIINFEKL (p < 0.05; Fig 6A).

We sought to characterize the protective efficacy of these antigen-specific CD8^+^ T cells by challenging vaccinated mice with *Lm-*OVA (10^5^ CFU) 11 days post vaccination. Plaque assays from spleen on day 2 following *Lm-*OVA challenge showed significant differences in bacterial load counts compared to controls. Ad5 vaccinated mice with HD5 co-administration led to a 5.7-log increase in bacterial loads (p < 0.01; Fig 6B), and the lack of protection in the Ad5 + HD5 group correlated to the reduction of SIINFEKL^+^ CD8^+^ T cells. In contrast, Ad26 vaccinated mice that received HD5 co-administration exhibited a 6-log reduction in bacterial loads (p < 0.01; Fig 6B) compared to controls. We suspect that a threshold of SIINFEKL^+^ CD8^+^ T cells may be necessary for protection in this model, consistent with prior studies [25–27]. These data demonstrated that HD5 differentially suppressed and augmented antigen-specific protective CD8^+^ T cells elicited by Ad5 and Ad26 vaccine vectors, respectively.

**Fig 7:**
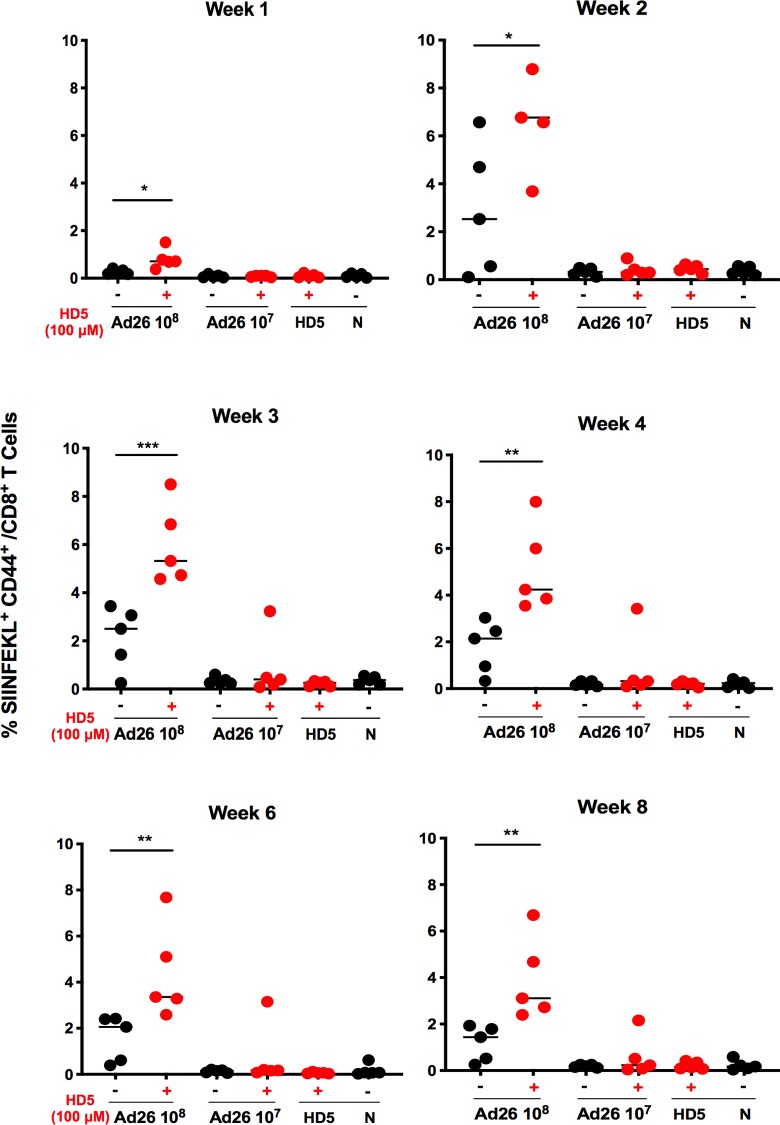
Longitudinal analysis of Ad26-elicicted antigen-specific CD8^+^ T cell responses. C57BL/6 mice (n = 5/group) were administered i.m. Ad26.SIINFEKL (10^8^ - 10^7^vp) pretreated with either PBS or 100 μM HD5. Mice were bled and SIINFEKL-specific CD8+ T cell responses were measured by H-2K^b^ tetramer staining at (A-F) weeks 1–8 post immunization. The data are representative of experiments performed two times. Mean ± SEM are shown. *** *p* < 0.001, ** *p* < 0.01, * *p* < 0.05, Mann-Whitney U Test (compared to virus only control).

Finally, we performed a longitudinal analysis to assess the durability of the effect of HD5 on antigen-specific CD8^+^ T cells elicited by Ad26. We immunized animals (n = 5/group) with Ad26.SIINFEKL (10^8^ or 10^7^ vp) and 100 μM HD5. Animals were bled at weeks 1, 2, 3, 4, 6, and 8 post immunization, and CD8^+^ T cell responses specific for SIINFEKL were evaluated by tetramer binding assays. Mice showed a peak 2.7-fold enhancement at week 2 (p < 0.05), and a 2.2-fold enhancement by week 8 (p < 0.01) of SIINFEKL^+^ CD8^+^ T cells compared to controls (Fig 7), demonstrating that HD5 enhanced Ad26-elicited CD8^+^ T cell responses for at least 8 weeks.

## Discussion

The ability of α-defensins to modulate innate and adaptive immune profiles induced by Ad vaccine vectors *in vivo* remains poorly characterized. Here we demonstrate that HD5 differentially modulates the immunogenicity and protective efficacy of Ad5 and Ad26 vectors in mice in a species-specific manner. HD5 augmented innate, cellular and humoral immune responses elicited by Ad26 and improved protective efficacy against a recombinant Listeria challenge. In contrast, Ad5-elicited immune responses and protective efficacy were dampened by HD5. Taken together, these findings show that HD5 can have different effects on different subtype Ad vectors *in vivo*.

Prior studies have shown that Ad differential susceptibility to α-defensins can be divided in two categories based on whether α-defensins suppress or enhance Ad vector expression [5,6]. These studies showed *in vitro* that Ad sensitivity to α-defensins is largely dependent on species specificity, and that adenovirus serotypes 19, 23, 25, 37, 51 (species D) and 41 (species F) are uniquely enhanced by α-defensins, whereas other Ad species show inhibition. Our *in vitro* data confirm and extend these observations. Furthermore, we identified two additional species D serotypes, Ad26 and Ad48 that displayed enhanced expression in the presence of HD5. We also showed that surface hexon hypervariable regions contribute to the HD5-augmented phenotype exhibited by Ad5HVR48. However, relatively few prior studies have explored the ability of α-defensins to modulate the immunogenicity of adenovirus vectors in vivo [8,28]. Our data showed enhanced levels of innate cytokines and chemokines, enhanced antigen-specific serum IgG responses, and an enhanced protective capacity of antigen-specific CD8+ T cells in a Listeria challenge model *in vivo* with Ad26 in combination with HD5 as compared to Ad26 alone. In contrast, Ad5-elicited immunogenicity was suppressed by co-administration of HD5. Our data are consistent with previous studies that have shown that α-defensins can modulate innate immunity [4,29] and thereby enhance specific serum IgG antibody responses [30,31] as well as enhance CD8^+^ T cell responses in mice [31,32].

Ad vectors can be influenced in many ways by host antiviral responses. It is believed that HD5 exhibits antiviral activity that prevents non-species D or F Ad vectors from escaping the endosome during intracellular trafficking [6]. Although the mechanism of enhancement of species D and F Ad vectors is not fully understood, it has been proposed that non-neutralizing HD5 binding epitopes on the capsid surface mask the electronegative surface charge and facilitate entry into cells, similar to the use of polycation formulations, which enhance adenovirus uptake and subsequent transgene expression *in vitro* [33–38]. Recently, Vragniau et al. [17] discovered a new mechanism used by a clade B, replication-competent Ad3 vector to overcome the antiviral effect of HD5 *in vitro*. Specifically, Ad3 produces subviral penton-dodecahedral particles that act as decoys for HD5, thus preventing the inactivation of progeny virus. These results may be due to the fact that a replication-competent virus was used in this particular case, whereas most other Ad-HD5 studies involve replication-defective viruses. Nonetheless, our study and others show that α-defensins have a significant impact modulating expression by Ad vectors and that more basic biology research is needed to fully elucidate these interactions.

Collectively, our data suggest that Ad vector-elicited immune responses are modulated by HD5 in a species-specific manner. The data presented here fills a knowledge gap by showing that HD5 has a profound impact on Ad vector-elicited innate, cellular, and adaptive immune responses *in vivo*. Such knowledge might facilitate the idea of using HD5 therapeutically to inhibit Ad types sensitive to HD5 like Ad5, but can also be explored for adjuvant discovery with Ad types similar to Ad26. To explore these effects to their full potential, further studies are warranted to define the specific mechanism of action of α-defensins on Ad-elicited adaptive immune responses, as well as the roles of the various cytokines and chemokines in modulating transgene-specific immune responses.

## Materials and methods

### Ethics statement

All animal studies were approved by the Beth Israel Deaconess Medical Center (BIDMC) Institutional Animal Care and Use Committee (IACUC), protocol # 005–2015. The BIDMC IACUC abides by the Office of Laboratory Welfare (OLAW) and United States Department of Agriculture (USDA) regulations and guidelines, and meets NIH standards set forth in the "Policy on Humane Care of Vertebrate Animals Used in Testing, Research, and Training" and the "Guidelines for the Care and Use of Laboratory Animals" (DHHS publication #NIH 85–23).

### Viruses

Replication-incompetent, E1/E3-deleted Ad5, Ad26, Ad35, Ad48 and Ad5HVR48 vectors expressing enhanced green fluorescent protein (eGFP), SIV_mac239_ (SIV Env), luciferase (luc), or the ovalbumin immunodominant epitope (SIINFEKL) were as previously described [16]. Briefly, the plasmid/cosmid system for these vectors consisted of a pAdApt adaptor plasmid consisting of the left ITR; the packaging signal; an expression cassette involving a cytomegalovirus promoter, a multiple cloning site for insertion of a trans gene, and the simian virus 40 polyadenylation transcription termination signal; and a 2.0–2.5-kb fragment downstream of the E1 region that enables homologous recombination with pWE cosmids in E1-complementing cells such as PER.C6. A pWE cosmid containing the majority of each respective Ad genome spanning from the pIX sequence to the right inverted terminal repeat (ITR) with a deletion of the E3 region and a modified E4 open reading frame 6 (E4orf6) sequence was used for adenovirus construction. Viruses were grown in PER.C6 cells and maintained until virus cytopathic effect was observed. The vectors were plaque-purified, analyzed for transgene expression, amplified in 24 triple-layer T175 flasks, purified by double CsCl gradient ultracentrifugation, and dialyzed into phosphate-buffered saline (PBS) containing 5% sucrose. Purified rAd vectors were stored at -80°C. Virus particle (vp) titers were determined by spectrophotometry [17]. Specific infectivity was assessed by PFU assays.

### Cells and peptides

A549 cells (ATCC) were passaged in Dulbecco’s modified Eagle’s medium (DMEM) supplemented with 10% fetal calf serum (FCS) and grown at 37°C and 10% CO_2_.

Synthetic HD5 (ATCYCRTGRCATRESLSGVCEISGRLYRLCCR; disulfide bonds between Cys3-Cys31, Cys5-Cys20, and Cys10-Cys30) and mutant HD5 (mHD5; ATSYSRTGRSATRESLSGVSEISGRLYRLSSR) were purchased from Atlantic Peptides, LLC (Lewisburg, PA) at >95% purity and reconstituted in sterile water to 5 mg/ml and stored at -80°C. Working stocks were diluted in phosphate-buffered saline (PBS, Life Technologies) to the desired concentration.

### Mice

Six- to ten-week-old BALB/C and C57BL/6 mice were purchased from the Jackson Laboratory (Bar Harbor, ME). Animals were immunized intramuscularly (i.m.) in the quadriceps with virus, virus pretreated on ice for 1 h with HD5/mHD5, or PBS control in a volume of 100 μl divided equally between both legs unless noted otherwise. Experiments were conducted with n = 5 mice per group unless noted otherwise. All animal experiments were performed with approval from the Beth Israel Deaconess Medical Center Institutional Animal Care and Use Committee.

### Transduction assays *in vitro*

Transduction assays were conducted to determine the effect of HD5 or mHD5 on eGFP expression by Ad5, Ad26, Ad35, and Ad48 *in vitro*. A549 cells were seeded at a concentration of 5.0X10^4^ in 24-well tissue culture plates the night before. The next day viruses were incubated with increasing concentrations of HD5 or mHD5 in serum-free DMEM and incubated on ice for 1 h. The mixture was then added to A549 cells that were pre-washed twice with 1X PBS to remove serum from the media. After a 2 h incubation at 37°C, the media and unbound virus was aspirated and the cells were cultured in complete A549 media for 24 h at 37°C. The next day the cells were washed twice with 1X DPBS, trypsinized, rinsed with AUTOMACS buffer (Myltenyi Biotec), and centrifuged at 1700 RPM (Sorvall Legend RT) for 5 min. Buffer was aspirated and cells were resuspended in 2% formaldehyde. Fixed cells were acquired on a LSR II flow cytometer (BD Biosciences) and analyzed for eGFP expression using FlowJo analysis software (Tree Star). Data are expressed as a percent of control infection in the absence of HD5. Virus concentrations were chosen that typically yielded 50% positive cells in control samples.

To analyze luciferase expression by Ad5, Ad48, and Ad5HVR48, A549 cells (1.0X10^4^ cells/well) cultivated in 96-well plates were infected with the indicated viruses at 2500 viral particles vp/cell to obtain a medium level of luminescence based on the detection limits of the instrument. After 2 hours, the infection medium was replaced with complete A549 medium. Culture supernatant was discarded and replaced with DPBS buffer 1 day post-infection. Steady Glo mix (100 μl) (Promega) was added into each well and incubated for 15 minutes at room temperature. Steady Glo/DPBS mix (100 ul) was transferred into new Black/White Isolaplate-96 plate and luminescence was detected by PerkinElmer 1420 multilabel reader. All experiments were performed in triplicate.

### *In vivo* animal imaging

BALB/C mice were used due to their white coat color to prevent less non-specific background signal compared to other mouse strains. Animals were anesthetized with 2% isoflurane and oxygen for hair removal and imaged as previously described [39–41]. Briefly, hair was removed from the left hind leg on the ventral posterior area of the mouse. Mice were immunized in the left hind leg with Ad5.luc (10^9^ vp) or Ad26.luc (10^9^ vp) with and without increasing concentrations of HD5 or 100 μM mHD5. Animals were injected intraperitoneally (i.p.) with 150 μl of 30 mg/ml D-luciferin substrate (Caliper Life Sciences) according to the manufacturer’s protocol. Luminescence was quantitated at 6 h, 1 day, 3 days, and 7 days post-immunization in an IVIS Lumina II charge-coupled device imaging system and Living Image software (Caliper Life Sciences). Images were integrated for 3 min, f/stop was 1.2, and binning was large.

### Luminex

C57BL/6 mice were immunized i.m. with empty Ad5 (10^10^ vp) and Ad26 (10^10^ vp) vectors that were pretreated with 100 μM HD5 or PBS for 1 h on ice. Seven hours post immunization, animals were bled, and sera were isolated from whole blood. Sera were treated with 0.05% Tween-20 (Sigma) in 1X DPBS (Life Technologies) for 15 minutes at room temperature and then evaluated using the Millipore Milliplex Map Mouse Cytokine/Chemokine Magnetic Bead Panel (Millipore, Billerica, MA) according to the manufacturer’s instructions. Samples were subsequently fixed with 4% formaldehyde in 1X DPBS for 1 h at room temperature, washed, and resuspended in Drive fluid (Luminex Corporation). Data were acquired on a MAGPIX running xPONENT version 4.2 software (Luminex Corporation) and analyzed using a 5-parameter logistic model with a 80%-120% standard acceptance range. Cytokine and chemokine levels were analyzed using GraphPad Prism v6.03 (GraphPad Software, CA, USA) and averaged for each experimental condition. Values below the limit of quantification were set to the limit of quantification of the analyte for analysis purposes. Statistical significance was assessed with a Wilcox statistical test using R language.

### SIV Env IgG endpoint ELISA

C57BL/6 mice were immunized i.m. with Ad5.SIVEnv (10^9^ vp) and Ad26.SIVEnv vectors (108–10^6^ vp) expressing SIV Env. Vectors were pretreated with 100 μM HD5 or PBS for 1 h on ice. Animals were bled, and sera were isolated 14 and 28 days post immunization. Endpoint enzyme-link immunosorbent assays (ELISAs) were employed to detect for SIV.Env serum-specific IgG as described [42,43]. Briefly, ELISA plates were coated overnight with 5 μg of SIV_mac239_ gp140 Env. Plates were blocked for 4 h with PBS, 2% bovine serum albumin (BSA, Millipore Sigma), and 0.05% Tween 20 (Millipore Sigma). Mouse sera was serially diluted, added to ELISA plates, and incubated for 1 h. Sera was incubated for 1 h with peroxidase-conjugated, affinity-purified rabbit anti-mouse secondary antibody diluted 1:2000 (Jackson ImmunoResearch Laboratories). Absorbance was read on a Spectramax Plus ELISA plate reader using Softmax Pro 4.7.1 software (Molecular Devices). Positive titers were defined as the greatest serum dilution with OD > 2-fold above naïve negative control serum OD.

Two additional groups of animals (n = 35/group) were immunized with 10^6^ vp of Ad26 with and without 100 μM HD5. We analyzed day 24 and 28 post-immunization time points by Endpoint ELISA (see above) and assessed the data for significance using a two-sided Fisher Exact Test by making comparisons between virus + HD5 and virus only groups.

### Listeria challenge

C57BL/6 mice were immunized i.m. with Ad5.SIINFEKL (10^8^ vp) or Ad26.SIINFEKL (10^8^ vp) vectors expressing an immunodominant epitope from the ovalbumin protein, OVA_257-264_ (SIINFEKL), which has been previously shown to protect against recombinant *Listeria monocytogenes* expressing the ovalbumin protein (*Lm*OVA) [41]. Vectors were pretreated with 100 μM HD5 or PBS for 1 h on ice. Animals were bled 11 days post immunization and sera were stained for tetramer-specific CD8^+^ T cells using the MHC class I tetramer H-2K^b^ loaded with the immunodominant OVA_257-264_ (SIINFEKL) epitope from ovalbumin protein [44]. Animals were subsequently challenged with 1.0 X 10^5^ colony forming units (CFU) of *Lm*OVA by i.v injection. Each experimental condition was assayed for colony forming units by plating on BHI-agar plates as previously described [45].

### Longitudinal analysis of Ad26-elicited antigen-specific CD8 T cells

C57BL/6 mice were immunized i.m. with Ad26.SIINFEKL (10^8^–10^7^ vp) vectors pretreated with 100 μM HD5 or PBS for 1 h on ice. Animals were bled on weeks 1, 2, 3, 4, 6, and 8 post immunization and sera were stained for tetramer-specific CD8^+^ T cells using the MHC class I tetramer H-2K^b^ loaded with the immunodominant OVA_257-264_ (SIINFEKL) epitope from ovalbumin protein.
